# Automatic Segmentation of Pathological Glomerular Basement Membrane in Transmission Electron Microscopy Images with Random Forest Stacks

**DOI:** 10.1155/2019/1684218

**Published:** 2019-03-25

**Authors:** Lei Cao, YanMeng Lu, ChuangQuan Li, Wei Yang

**Affiliations:** ^1^School of Biomedical Engineering, Southern Medical University, GuangZhou 510515, China; ^2^Central Laboratory, Southern Medical University, GuangZhou 510515, China

## Abstract

Pathological classification through transmission electron microscopy (TEM) is essential for the diagnosis of certain nephropathy, and the changes of thickness in glomerular basement membrane (GBM) and presence of immune complex deposits in GBM are often used as diagnostic criteria. The automatic segmentation of the GBM on TEM images by computerized technology can provide clinicians with clear information about glomerular ultrastructural lesions. The GBM region on the TEM image is not only complicated and changeable in shape but also has a low contrast and wide distribution of grayscale. Consequently, extracting image features and obtaining excellent segmentation results are difficult. To address this problem, we introduce a random forest- (RF-) based machine learning method, namely, RF stacks (RFS), to realize automatic segmentation. Specifically, this work proposes a two-level integrated RFS that is more complicated than a one-level integrated RF to improve accuracy and generalization performance. The integrated strategies include training integration and testing integration. Training integration can derive a full-view RFS_1_ by simultaneously sampling several images of different grayscale ranges in the train phase. Testing integration can derive a zoom-view RFS_2_ by separately sampling the images of different grayscale ranges and integrating the results in the test phase. Experimental results illustrate that the proposed RFS can be used to automatically segment different morphologies and gray-level basement membranes. Future study on GBM thickness measurement and deposit identification will be based on this work.

## 1. Introduction

Primary glomerular disease is the most common renal disease in China [1]. The diagnosis of renal diseases is largely dependent on renal biopsy, which is regarded as the gold standard. Transmission electron microscopy (TEM) combined with optical microscopy and immunofluorescence examination constitutes a continuum of pathological diagnosis of renal diseases [2]. TEM allows the observation of pathological changes in the microstructure of various glomerular cells that cannot be resolved under an optical microscope. Thus observations from light and immune pathology can be verified at an ultrastructure level [3]. Studies have found that ultrastructural study provided fundamental or important diagnostic information for 44.3% of renal biopsies [4]. Therefore, TEM is essential for the diagnosis of certain nephropathy. Considering the complexity of the TEM image of the glomerulus and related lesions, it is time consuming and labor intensive for a pathologist to visually recognize subtle pathological changes, resulting in a huge workload. Nevertheless, after the initial screening of the computer, the diagnostic efficiency and accuracy of glomerular diseases can be improved with the help of automatic image-processing technology.

**Figure 1:**
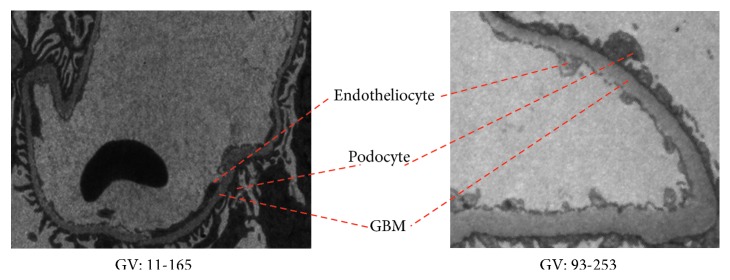
Interimage shape variations and intraimage grayscale inconsistency of GBM (GV: gray value).

The diagnosis of many renal diseases is closely related to the glomerular basement membrane (GBM) [5]. The basement membrane, along with the lining of the endotheliocytes and the lining of the podocytes on the outside, forms the filtration barrier, allowing the blood to filter out and form the urine, as shown in Figure 1. The changes of thickness in GBM and presence of immune complex deposits in GBM are often used as diagnostic criteria for certain nephropathy, such as membranous nephropathy with extensive membranous thickening and varying amounts of immune complexes, Alport syndrome with diffuse membranous thickening, and familial recurrent hematuria syndrome (thin basement membrane nephropathy) with diffuse thinning of GBM [6, 7]. Manual measurement of the thickness of GBM is an early auxiliary [8, 9], but the workload is very expensive. Then, some semiautomatic software tools [10, 11] are used to obtain the thickness of GBM more quickly and conveniently but still need manual intervention. In terms of morphological complexity, the autoidentification difficulty of deposits is the same as or even greater than GBM and literatures on this have not been found. In practicality, the thickness measurement and deposit identification can be realized subsequently and automatically on condition that the GBM region is completely autorecognized or is segmented.

Early in 1993, Ong et al. [12] applied adaptive window-based tracking to segment glomerular TEM images. Since then, a few semiautomatic or fully automatic methods have been proposed. Kamenetsky et al. [13] and Rangayyan et al. [14] achieved GBM segmentation and measurement through region division and dynamic contour modeling. Wu et al. [15] and Wu and Dikman [16] also proposed two methods. One is to obtain the center line of the GBM by interpolating manual mark points and then autosegment GBM through distance mapping and low-pass filtering [15]. Another method involves the use of threshold and morphological method with no manual mark [16]. Liu et al. not only segmented the GBM but also measured its length and counted the number of slits [17]. Most existing methods mentioned above have made some contribution, but there are still many problems unresolved. These methods either require tedious manual initialization that involves extra work for pathologists and introduces possible subjective errors, or they can only be used to segment truncated GBM fragments with increased contrast and single direction as showed in their experimental results. Therefore, ensuring segmentation quality for the whole complex GBM images remains challenging.

Two common difficulties associated with GBM segmentation are interimage shape variations and intraimage grayscale inconsistency. Figure 1 shows that the low contrast between the GBM segment and surrounding tissues, such as endotheliocytes and podocytes, and variations in the form and width of GBM segments cause difficulty in autoextracting features. In addition to the complex structure of a pathological section, the grayscale distribution of TEM images is very wide because of the uncertainty of sample prefabrication and the illumination inhomogeneity of transmission imaging.

To address the first challenge of autoextracting features, we employ a pixel-wise classifier, namely, random forest (RF) [18], based on machine learning to avoid relying on hand-crafted features. RF is a committee of weak learners (e.g., decision tree) to solve classification and regression problems without manually specifying some features through the construction and combination of multiple decision trees and random selection of attributes [19, 20], which can be used to cope with the complex structural characteristics of biological images. RF has been widely explored from medical image-processing fields, especially detection tasks, including early identification or prediction of Alzheimer's disease [21], adrenal gland abnormality detection [22], and automatic cardiac segmentation [23].

An enhanced generalization effect based on a single RF classifier is hardly obtained because of the grayscale inconsistency intraimage. To address this second challenge, we propose an RF stack (RFS) model based on a wider grayscale range of images. After assigning TEM images to different grayscale groups, we sample from all these groups and train a full-view RF classifier as RFS_1_ and multiple RF zoom-view classifiers as RFS_2_. In the segmentation phase, each pixel of the new GBM image is classified automatically through full-view and zoom-view RFS and the candidate segment results are combined and optimized. Thus, the segmentation accuracy is improved by using this two-level integrated machine learning method.

The remaining sections of this paper are organized as follows. In Section 2, information regarding GBM image selection and preprocessing is described and the details of the proposed RFS model for GBM segmentation is introduced. In Section 3, experimental results are reported and discussed. In Section 4, the conclusion is presented.

## 2. Materials and Methods

### 2.1. Data and Materials

#### 2.1.1. Image Data

Renal biopsy specimens were immediately fixed with 2.5% cold glutaraldehyde with 0.1 M phosphate buffer at pH 7.3 for 4 h, washed with phosphate buffer, postfixed with 1% osmium tetroxide in the same buffer, dehydrated with a graded series of ethanol, and embedded in Spurr resin. Ultrathin sections (70 nm) were contrast enhanced with uranyl acetate and lead citrate and examined using a Hitachi H-7500 electron microscope (Tokyo, Japan) at 60 kV. All of the sections were imaged with MORADA G3 (EMSIS Corporation of Japan) at 5000x magnification. In the field of vision, a whole glomerulus, including the glomerular capillaries and the basement membrane, was selected. Continuous filming was conducted using the attached digital imaging system and controlled by the Pathological Image Workstation of the NanFang Hospital in Guangzhou, China.

#### 2.1.2. Preprocessing of TEM Images

**Figure 2:**
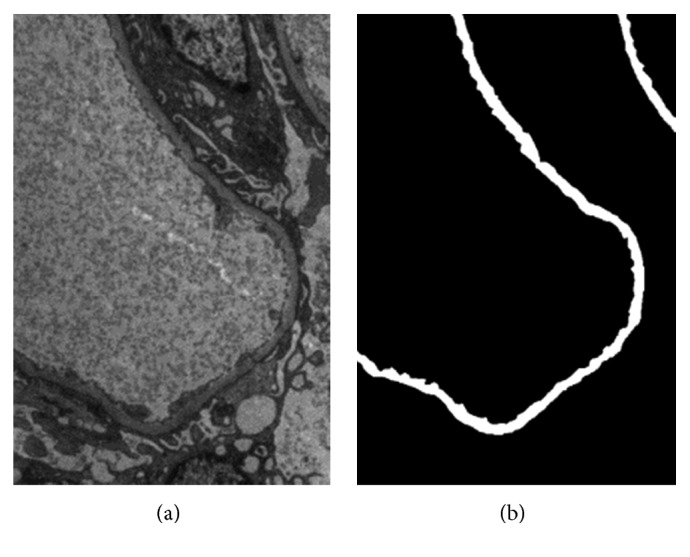
TEM image (a) and the corresponding binary mask image of GBM (b).

The pathologists collected 351 images from the obtained glomerular TEM images to build a GBM image database. Of these 351 images, 330 were used as a training set and divided into different groups (*N* = 37) according to the range of GBM's intensity. In the test set, 21 images with different sizes and various basement membrane types, such as stripe-, closed-, and compound-type, were used. The image pixels were divided into GBM and background.  Figure 2(a) shows the original TEM image, and Figure 2(b) illustrates its corresponding GBM binary mask manually labeled by the pathologist.

### 2.2. Method Overview

#### 2.2.1. Workflow Diagram

**Figure 3:**
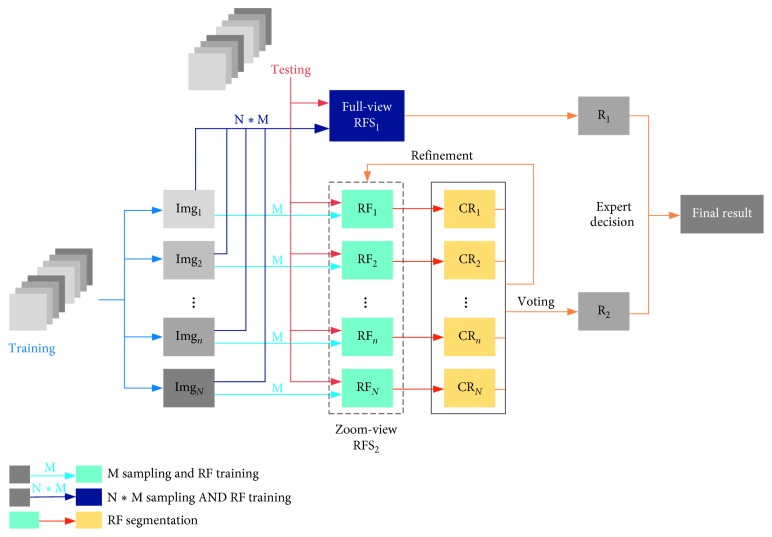
Flowchart of the proposed RFS method.

RF is an ensemble machine learning method, which can be applied to image segmentation by classifying pixels into target or background. The proposed RFS is a RF-based multilevel integrated structure that mainly involves two phases: hierarchical training and refinement testing. The implementation process of RFS is shown in Figure 3.

Train phase: from the prebuilt GBM database, an image is randomly selected from each image group with different GBM grayscale ranges. Hence, *N* images, namely, Img_1_ to Img_*N*_, needed for one training session are ready. The simultaneous sampling of *N* images and follow-up training yield RFS_1_ called the full-view RFS. Then, *N* RF classifiers, RF_*i*_(*i*=1,…, *N*), are generated by sampling and training each image individually, and RFS_2_, which is called zoom-view RFS, covering different grayscale ranges is constructed.

Test phase: each pixel of the test image is classified by RFS_1_ and constitutes a candidate segmentation *R*_1_. Each pixel of the test image is classified by each RF_*i*_ in RFS_2_ and got *N* coarse segmentation results, namely, CR_1_ to CR_*N*_. Then, another candidate segmentation *R*_2_ is obtained after an iterative refinement scheme. The final segmentation is selected from *R*_1_ and *R*_2_ by a human expert.

#### 2.2.2. Software Tools

**Figure 4:**
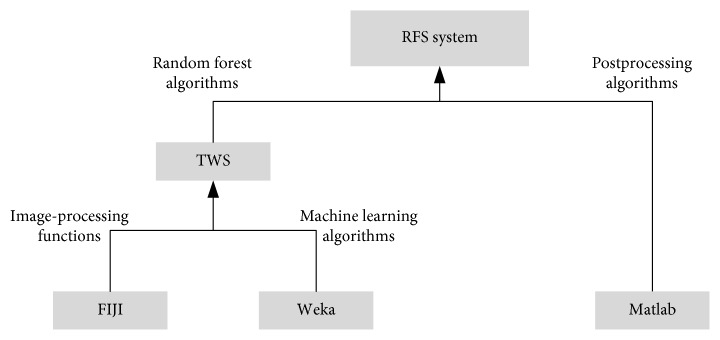
Software tools.

The image-processing and analysis software FIJI (ImageJ) is developed by the US National Health Administration, and FIJI-based secondary development is well known. In this paper, we selected a FIJI plug-in named Trainable Weka Segmentation (TWS) (http://imagej.net/Trainable_Weka_Segmentation) [24], which is based on the free open-source software Weka [25]. TWS combines a series of machine learning algorithms to perform pixel-based image segmentation.  Figure 4 shows that TWS is modified and integrated with some image-processing functions provided by Matlab to meet the needs of GBM image segmentation.

### 2.3. Training

#### 2.3.1. Random Forest

RF [23] is a common method for ensemble learning whose training algorithm relies on bagging integration and random attribute selection in the construction of the decision tree. The training of one RF is shown in a blue arrow line in Figure 3. Bootstrap sampling technology is used to generate *T* training subsets from the original training set, and *T* decision tree models are established to form one RF. An RF segmentation is illustrated in an orange arrow line in Figure 3. The test image is separately classified by the *T* decision trees in the RF, and the result of each decision tree is aggregated to the final output by voting. In this paper, we refer this to level 1 integration.

#### 2.3.2. RFS Classifiers

**Figure 5:**
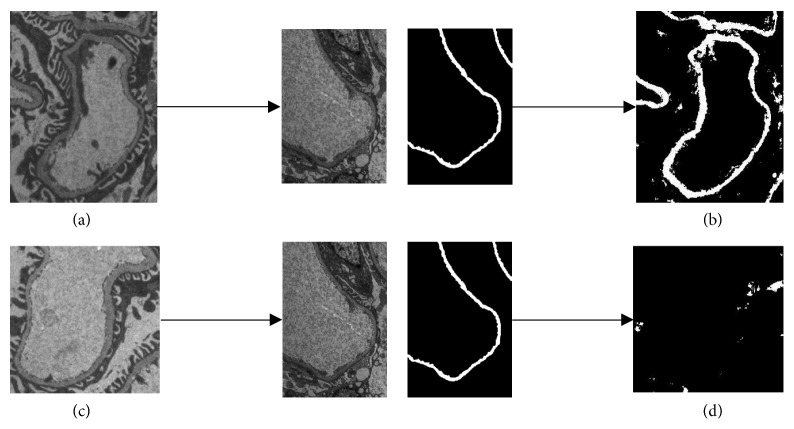
Segmentation results of TEM images with different grayscale ranges by using the same RF classifier.

Considering that the intensities of GBM in various TEM images are significantly different, a single RF classifier cannot extract different grayscale features of all TEM images and the segmentation performance is unstable. For example, given an RF classifier sampled and trained from Figure 2, the membrane illustrated in Figure 5(a) can be well segmented as shown in Figure 5(b) because of the similar grayscale of the GBM in Figure 5(a) and the classifier. A poor result is obtained by using the same classifier to segment the membrane in Figure 5(c), and the entire membrane fragment is almost not segmented as shown in Figure 5(d).

To address this problem, we introduced level 2 integration. We first assigned all training images to *N* groups according to the average intensity in the GBM regions. Then, RFSs were constructed. Full-view and zoom-view methods were proposed. The full-view method simultaneously takes samples from *N* different grayscale range images. As *M* pixels are sampled in each image, *M* × *N* pixels are sampled at the same time for training and RFS_1_ is obtained. RFS_1_ is a large file in the same logic form of an RF. Due to the large number of sampled pixels and the limited depth of the tree, we can assume that leaf nodes form stacks.

The zoom-view method separately takes samples from *N* grayscale range images for training and obtains a series of RFs ranging from RF_1_ to RF_*N*_, thereby forming RFS_2_. Since the sampling points of each forest in RFS_2_ are well targeted to images with the similar range, the generalization performance of a single forest is limited and the results need to be integrated and refined at the test or segment phase.

#### 2.3.3. Implementation Details

In the prebuilt GBM database, the TEM images in the training set were divided into *N*=37 groups according to the average intensity of GBM. The average intensities of the GBM begin from about 73 Hounsfield units with an intensity step of 3 Hounsfield units.

TWS has 15 applicable features, and 14 of them were selected as the inputs of the decision tree in this paper: common grayscale features (mean, minimum, maximum, median, and variance), boundary features (Sobel filter, Hessian, and difference of Gaussians), texture features (Gaussian blur, entropy, and Kuwahara filter), and other features (membrane projections, Lipschitz filter, and neighbors).

Other RF training parameters include the number of decision trees (*T*=100), the depth of decision trees (*D*=9), and the number of sampling points per image (*M*=2000). The selection of some parameters is discussed in Section 4.

### 2.4. Segmentation

Given an image to be segmented or tested, two candidate segmentation results can be separately obtained by RFS_1_ and RFS_2_. Pixel-by-pixel classification through full-view RFS_1_ yields a candidate segmentation *R*_1_. The process of getting candidate *R*_2_ is more complicated. After the preparation of a series of coarse results, namely, CR_1_ to CR_*N*_, dealt by zoom-view RFS_2_, the probability map are reconstructed and the candidate *R*_2_ can be obtained after postprocessing and iterative refinement is completed. Finally, *R*_1_ and *R*_2_ are evaluated by experts to determine an enhanced segmentation.

#### 2.4.1. Probability Map

For each image pixel to be segmented, equation (1) is used to reconstruct the probability map with the *N* coarse segmentation results, namely, CR_1_ to CR_*N*_:

(1) $$\begin{array}{c} p\left(i,j\right)=\frac{n\left(i.j\right)}{N}, \end{array}$$

where *N*=37 is the total number of coarse segmentation results, *n*(*i*, *j*) is the frequency of the pixel of *i*th row, *j*th column is marked as GBM by each CR, and *p*(*i*, *j*) is the probability of the pixel (*i*, *j*) as GBM.

#### 2.4.2. Postprocessing

In the probability map, a large gray value of a pixel corresponds to a high probability to become GBM. Therefore, by maximizing the similarity belonging to the same category or avoiding it to reach the minimum, the fuzzy C-means (FCM) [26] algorithm is utilized for postprocessing to divide the image into the GBM regions and the background. Then, after some false positives are removed through a morphological operation, the GBM regions can be extracted.

#### 2.4.3. Iterative Refinement

Not every coarse segmentation results CR_*i*_(*i*=1,…, *N*) obtained from RFS_2_ provides useful information on the construction of the probability graph. In some extreme cases, some coarse segmentation results are counterproductive to the probability map. Therefore, a refinement process is described in Algorithm 1.

**Figure 6:**
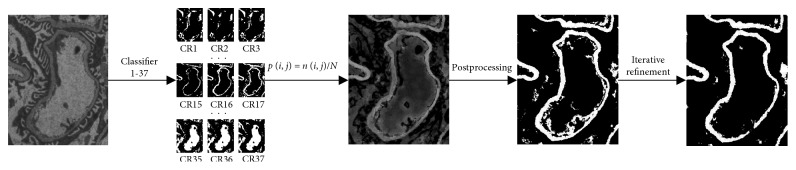
Segmentation process of RFS_2_.

Figure 6 shows the whole segmentation process of RFS_2_, including (1) 37 RF classifiers, (2) probability map, (3) postprocessing, and (4) iterative refinement.

#### 2.4.4. Manual Interaction for Final Decision

For a test image, the candidate segmentation results *R*_1_ and *R*_2_ are compared with a gold standard labeled by a pathologist. For new images without gold standard, a user can compare *R*_1_ with *R*_2_ and make the final decision based on the following aspects:

1. Whether the foot process of the epithelial cell or the cytoplasm of the endothelial cell is inappropriately contained in the region of the basement membrane because its electron density is similar to that of the basement membrane
2. Whether the subepithelial immune deposit is erroneously excluded from the basement membrane because its electron density is higher than that of basement membrane
3. The continuity of the basement membrane should be cautiously analyzed because pathological fracture defects of the basement membrane are few

### 2.5. Evaluation Metrics

The accuracy of the proposed method is evaluated by Jaccard coefficient, which is widely utilized to evaluate the performance of segmentation methods [27–29]. It is a measure of geometric similarity defined by

(2) $$\begin{array}{c} \text{Jaccard}\left(A,B\right)=\frac{A\;\cap \;B}{A\;\cup \;B}, \end{array}$$

where *A* and *B* are the results of manual segmentation by human experts and the proposed method. The range of Jaccard value is [0, 1]. A large coefficient value corresponds to an accurate segmentation result.

## 3. Experiments and Results

In this study, 21 TEM images with different grayscale ranges, sizes, and basement membrane morphologies are used for evaluation. These images are manually segmented by pathologists as the gold standard.

### 3.1. Validation

**Figure 7:**
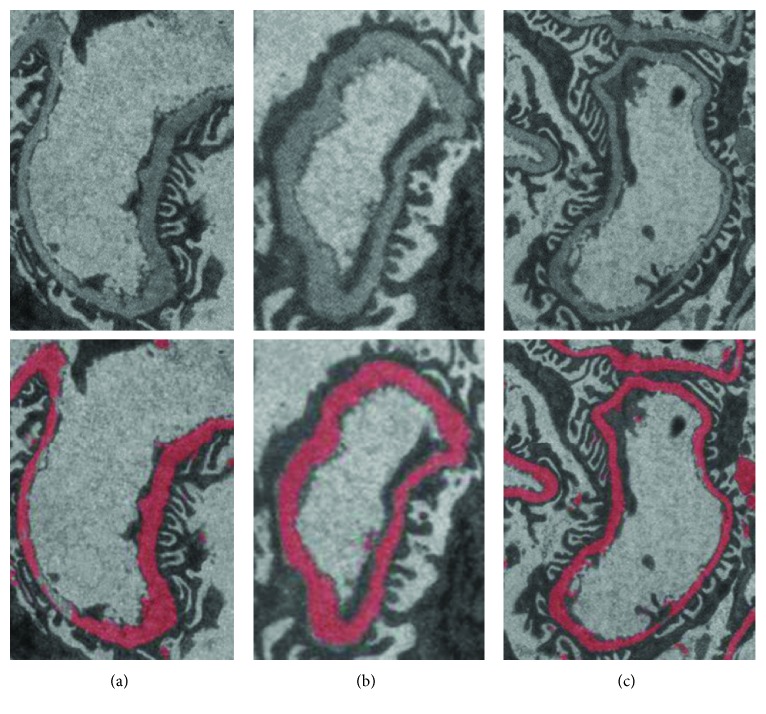
Segmentation results of RFS with different morphologies of GBM. (a) Strip type: size, 217∗307; Jaccard, 0.75. (b) Close type: size, 150∗206; Jaccard, 0.84. (c) Compound type: size, 282∗367; Jaccard, 0.76.

The RFS method provides robust segmentation results of GBMs with different morphologies and grayscale ranges. RFS_1_ and RFS_2_ are trained with *M*=2000 and *N*=37.  Figure 7 shows the segmented images obtained from the strip-, closed-, and compound-type basement membranes by using the RFS method. The top line shows original images, and the bottom line shows the corresponding segmentation results. As can be seen from the figure, although the orientation, width, and other morphologies of the GBM vary greatly, the results of the segmentation are relatively accurate. For the three test images shown, the Jaccard values are higher than 0.75.

**Figure 8:**
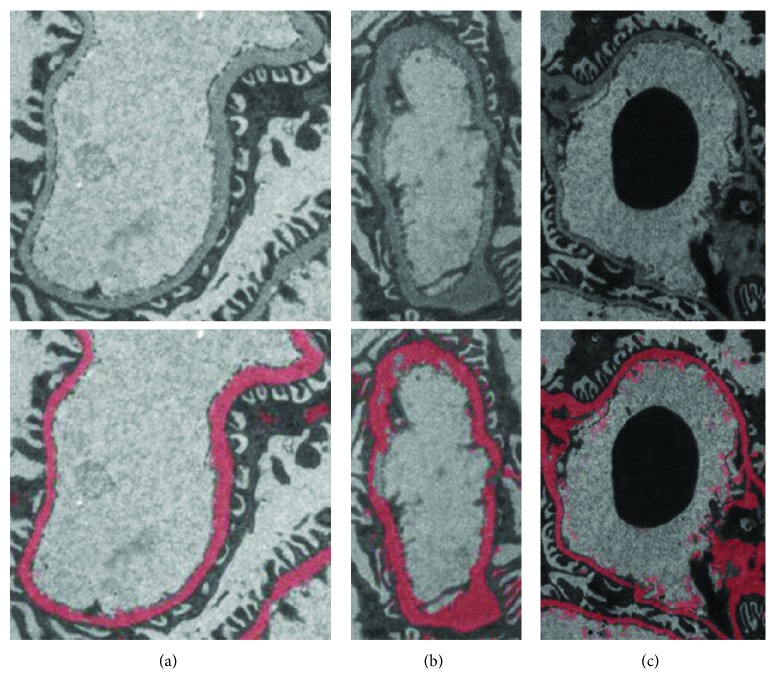
Segmentation results of RFS with various grayscale ranges of GBM (RoGV: range of gray value). (a) RoGV: 54–255; size: 282∗274; Jaccard: 0.70. (b) RoGV: 43–187; size: 168∗308; Jaccard: 0.71. (c) RoGV: 12–167; size: 297∗408; Jaccard: 0.65.

Figure 8 shows the segmented images from different grayscale range basement membranes. It can be seen that most of the GBM is accurately segmented. Compared with RF, the segmentation results of RFS are better. For example, the original TEM image shown in Figure 5(c) fails with RF segmentation, but it can be well segmented by RFS, as shown in column 1 of Figure 8.

As shown in Figures 7 and 8, although the morphology and grayscale range of the basement membrane vary greatly, the results of the RFS segmentation are stable, indicating a good generalization performance of the RFS method. Future study on GBM thickness measurement and deposit identification will be based on this.

### 3.2. Influence of the RF Classifiers of Different Grayscale Ranges

**Figure 9:**
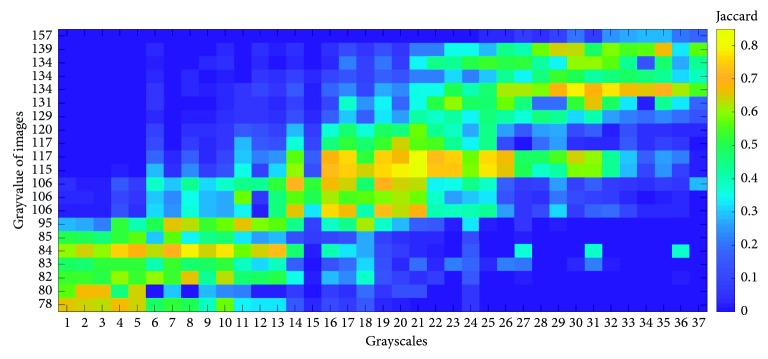
Heat map of segmentation results of different grayscale ranges of RF classifiers.

A multilevel integrated RFS classifier is constructed to address the generalization problem of GBM segmentation. This is based on the hypothesis that, for an RF classifier, the closer the grayscale range of the image to be segmented is to the training image, the better the segmentation effect will be. The experimental results from the heat map in Figure 9 confirm this hypothesis. The horizontal axis from low to high represents 37 RF classifiers corresponding to 37 grayscale ranges of the training images. The range of the 1st grayscale is [73.5, 75.5], and the range of the 37th grayscale is [145.5, 147.5]. The vertical axis represents the grayscale mean value of the GBM of the 21 test images. The grayscale mean value of test image 1 is 78, which is close to the grayscale range of levels 1 and 2. The 21st test image has an average grayscale of 145, which is higher than the grayscale range of level 37.

In this experiment, each test image is separately segmented by these 37 RF classifiers of different grayscale ranges, and the corresponding Jaccard value is shown in different colors. An accurate segmentation result corresponds to a high Jaccard value, and its color turns to bright yellow. It can be seen from the color distribution in Figure 9 that most RF classifiers are only sensitive to test images with a close grayscale range. However, as shown in Figures 7 and 8, the RFS can accurately segment test images with different grayscale ranges that means the generalization performance of RF is not good as proposed RFS.

### 3.3. Differences between Full-View RFS_1_ and Zoom-View RFS_2_

**Figure 10:**
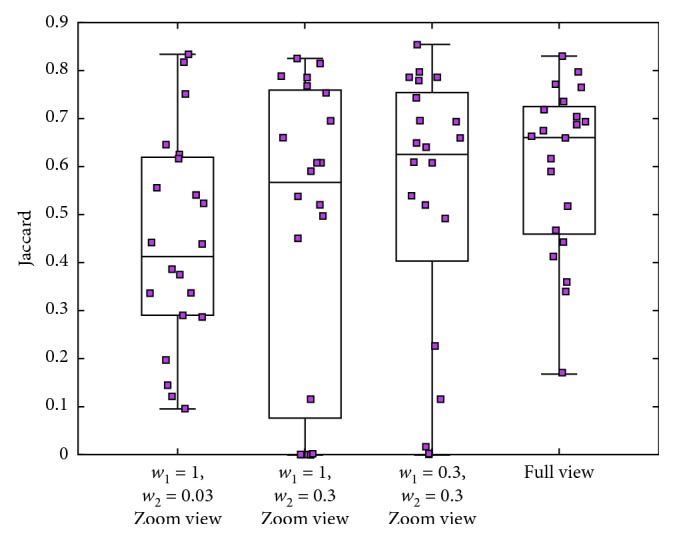
Jaccard values of zoom-view RFS_2_ at three different parameter combinations and full-view RFS_1_.

The multilevel RFS is constructed in full view and zoom view as shown in Figure 3. The refinement details of zoom-view RFS_2_ are given in Algorithm1, involving the setting of thresholds *w*_1_ and *w*_2_. The Jaccard values of the segmentation result for each test image (the pink dots) by using RFS_2_ with three different parameter combinations are shown in columns 1, 2, and 3 of Figure 10, where *w*_1_=1 indicates that all *N*=37 coarse segmentation results are included in the steps of iterative optimization without filtering. The Jaccard values of the segmentation result by using full-view RFS_1_ are shown in column 4 of Figure 10.

Figure 10 shows that the full-view RFS_1_ is more robust than the zoom-view RFS_2_. Regardless of how thresholds *w*_1_ and *w*_2_ are set, the mean value of RFS_2_ is lower than RFS_1_. However, when *w*_2_=0.3, the segmentation result of some test images of RFS_2_ is better than that of RFS_1_. In the experimental data, the maximum segmentation accuracy is 0.85 but the minimum value is almost 0 and all these values are obtained through RFS_2_.

The stability of RFS_1_ is mainly caused by a large sample training of the classifier, involving 2000 × 37 sample points. However, the disadvantage of this method is its high-intensity computation. Some low accuracy of RFS_2_ is caused by the effect of *N* course segmentation results on refinement process. If the similarity of most results with the gold standard is insufficient, a poor optimized image is obtained. Otherwise, the result is enhanced or even exceed that of RFS_1_. Therefore, the final step of this method involves the selection between the two candidate segmentation results of RFS_1_ and RFS_2_.

### 3.4. Effects of Postprocessing and Refinement

**Figure 11:**
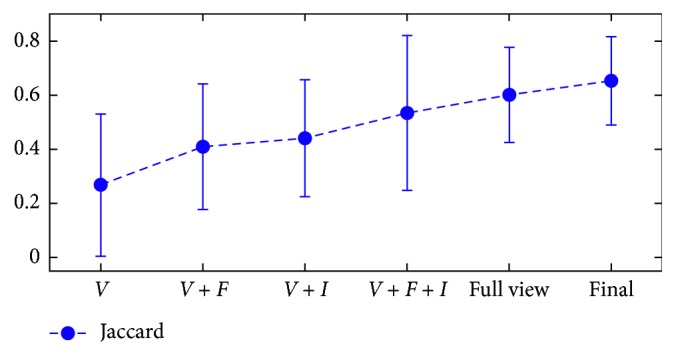
Effects of postprocessing and refinement methods.

The following methods are adopted to validate the effect of postprocessing and iterative refinement on RFS_2_: (1) voting method (*V*), (2) additional postprocessing with FCM on the voting result (*V* + *F*), (3) additional iterative refinement on the voting result (*V* + *I*), and (4) additional iterative refinement on method 2 (*V* + *F* + *I*), namely, RFS_2_.  Figure 11 shows the mean and variance of methods (1)–(4), full-view RFS_1_ (5), and the final result (6). The mean accuracy of *V* is the lowest among them. *V* increases after FCM postprocessing and further improves after iterative refinement is completed. The final result includes the maximum mean and a relatively small variance.

## 4. Discussion

### 4.1. Methods for Constructing Ensembles

Ensemble methods construct a set of classifiers and then classify new data points by taking a weighted vote of their predictions. Dietterich [30] assumed that five general purpose ensemble methods exist: enumeration of hypotheses, manipulation of training examples, manipulation of input features, manipulation of output targets, and injection of randomness. We adopted two of them and developed their corresponding RFS methods. Full-view RFS_2_ manipulates the training examples to generate multiple hypotheses. Considering the complicated GBM images, we sample multiple grayscale images to further increase the diversity of the hypotheses. Zoom-view RFS_1_ manipulates the output probability graph to achieve integration. The iterative refinement step is addressed to reduce the adverse effect of the rough segmentation result, further improving the accuracy of segmentation.

### 4.2. Selection of the Parameters

The number of decision trees is among the most important parameter in the application of RF algorithm in medical image segmentation [31]. Theoretically, with the increasing number of decision trees, the classification accuracy of the algorithm gradually increases as computational cost rapidly increases. The optimal number of trees should obtain a good balance between evaluation metric, processing time, and memory usage. In this study, the number of decision trees is experimentally set to 100.

The number of sampling points is another critical parameter in the RFS method. In our experiment, as the number of sampling points increases from 200 to 2000 per training image, the accuracy rate of the RFS classifier increases by approximately 10%, whereas the accuracy is not improved greatly if the number of sampling points continuously increases. Thus, the total sampling point is set to 74,000, where *M*=2000 and *N*=37, to obtain the best result.

**Figure 12:**
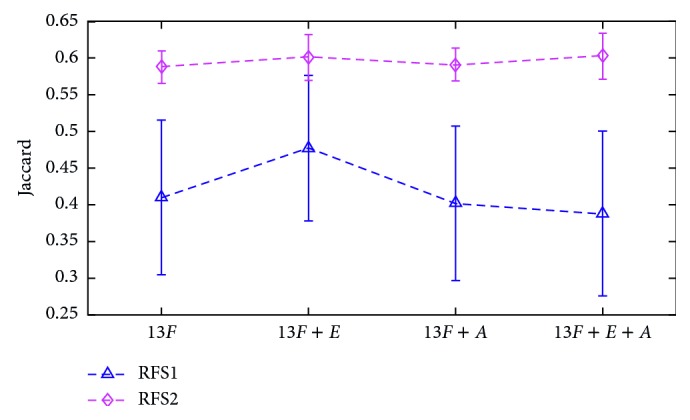
Effects of different image features with or without entropy and anisotropic diffusion.

In TWS, 15 available image feature attributes are provided in the decision tree construction. In our experiment, the application of most features can improve the accuracy of segmentation, but entropy (*E*) and anisotropic diffusion (*A*) are time consuming. Let 13*F* indicates 13 other features aside from *E* and *A*; Figure 12 shows that the application of feature *A* not only costs more time but also reduces the accuracy of segmentation whether by using classifier RFS_1_ or RFS_2_. Therefore, only 14 features other than anisotropic diffusion are used to construct the decision tree in the proposed RFS method.

### 4.3. Limitations of the Proposed Method

Our experiment results reveal that the proposed RFS method obtains poor performance for some cases. For example, for a low-contrast image, the accuracy rate of voting is almost 0. Only the accuracy rate of *V* + *I* reaches 23%, whereas the accuracy rates of the other methods are below 20%, even that of RFS_1_. Such bad results greatly reduce the average accuracy of the RFS method. RF can be combined with other pattern recognition methods for better performance. Lu et al. [32] applied incomplete RF with a robust vector machine for the early identification of mild cognitive impairment. This method outperforms two other semisupervised learning methods. Therefore, to improve the segmentation accuracy of low-contrast GBM images, the combination of RF methods with other methods will be our future work.

## 5. Conclusion

The segmentation of the whole GBM region in TEM pathological images can provide more rapid and intuitionistic observation for the morphological change and can reduce the tedious and expensive manual workload of the pathologist. This work proposed a two-level integrated RFS method involving training integration and testing integration to autosegment a GBM image. A total of 351 clinical images were included in the experiment. The accuracy and generalization ability of the RFS method were validated. Experimental results illustrated that the proposed method could be used for the automatic segmentation of GBM with different morphological characteristics and grayscale ranges. Further study is underway to improve segmentation accuracy of the automated CAD system and to implement GBM thickness measurement and deposit autorecognition for auxiliary pathological diagnosis.

## Figures and Tables

**Algorithm 1:**
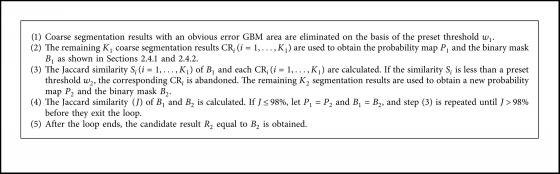


## Data Availability

The image data used to support the findings of this study are available from the corresponding author upon request.
